# Production of Carbamic Acid Dimer from Ammonia-Carbon Dioxide Ices: Matching Observed and Computed IR Spectra

**DOI:** 10.3390/life9020034

**Published:** 2019-04-23

**Authors:** Zikri Altun, Erdi Bleda, Carl Trindle

**Affiliations:** 1 Physics Department, Marmara University, 34722 Istanbul, Turkey; altunzikri@gmail.com (Z.A.); erdia@g.clemson.edu (E.B.); 2 Chemistry Department, University of Virginia, Charlottesville, VA 22902, USA

**Keywords:** prebiotic chemistry, carbamic acid, carbon dioxide-ammonia ices, infrared spectra, anharmonicity

## Abstract

The production of complex molecules in ammonia–carbon dioxide ices is presumed to pass through species of formula H_3_N:CO_2_ with further addition of ammonia and carbon dioxide. One possible landmark, carbamic acid, H_2_NCOOH, has been implicated among the products of warming and irradiation of such ices. Experimental study of the IR spectra of residues has suggested the presence of related species, including weakly bound 1:1 and 2:1 complexes of ammonia with carbon dioxide, zwitterionic carbamic acid, ammonium carbamate, and the dimer of carbamic acid. We computed the energetics and vibrational spectra of these species as well as the complex between ammonia and carbamic acid for gas and condensed phases. By means of a new spectrum-matching scoring between computed and observed vibrational spectra, we infer species that are most probably present. The leading candidates are ammonium carbamate, the carbamic acid–ammonia complex, and the carbamic acid dimer.

## 1. Experimental Evidence for Carbamic Acid

The evolution of complex and perhaps biologically relevant molecules from the simple molecules well established to exist in the interstellar medium is a central issue in astrochemistry [1]. Among the species of small molecules containing C, H, N, and O that may be formed on grains in ices are variants of carbamic acid, H_2_NCOOH. Such species can be precursors of biologically significant species including amino acids. Existence of a T-shaped 1:1 complex of ammonia with carbon dioxide was inferred from molecular beam studies by Fraser et al. [2]. Terlouw and Schwarz [3] showed by mass spectrometric means that carbamic acid could be produced in the gas phase. Frasco [4] and Hisatsune [5] inferred from IR spectroscopy of the residue from VUV-irradiated and warmed ammonia–carbon dioxide ices that ammonium carbamate NH_4_(+)H_2_NCOO(−) is formed in the solid. Chen et al. [6] irradiated ammonia–carbon dioxide–water ice with 4–20 eV photons, and monitored the 250 K residue by IR. They assigned certain features of the spectra to non-zwitterionic H_2_NCOOH.

Bossa et al. [7] interpreted the IR spectrum of low-temperature ammonia–carbon dioxide ices as suggesting the presence of the 1:1 and 2:1 complexes of ammonia with carbon dioxide, and modeled the structures by DFT calculations. In a separate investigation, Bossa et al. [8] warmed 1:1 ammonia–carbon dioxide ice from 10 K to 260 K and monitored the residue by FTIR and mass spectrometry. A species Bossa et al. [8] identified as ammonium carbamate decomposes to ammonia and carbon dioxide above 220 K. Other signals emerged in their study that they associate with carbamic acid, carbamic acid dimer, and ammonium carbamate.

Rodriguez-Lazcano et al. [9] studied solid mixtures of ammonia and carbon monoxide by vapor deposition and also hyperquenching on a cold plate with and without water, over a T range of 120–240 K. They report that ammonium carbamate is the primary product but that carbamic acid species are formed as well; in the presence of water, ammonium bicarbonate is also detected.

Noble and co-workers [10] studied the kinetics of production of ammonium carbamate over a temperature range of 70–90 K. Carbamic acid was proposed as an intermediate; several IR features were attributed to carbamic acid and its dimer.

Irradiation of model ices with high energy electrons or ions is of long-standing interest. Berit and co-workers [11] irradiated carbon dioxide–ammonia ices with 30 keV beams of He cation, but identification of species produced by the beam was difficult owing to intense absorption by the abundant ammonia, carbon dioxide, and (synthesized) water. Khanna and Moore [12] irradiated a composite solid composed of a layer of ammonia below a layer of carbon dioxide–water ice with 1 MeV protons; IR analysis of the 250 K sublimate suggested the presence of zwitterionic H_3_N(+)-COO(−) carbamic acid. Jheeta et al. [13] irradiated ammonia–carbon dioxide ices with 1 keV electrons, and inferred the production of ammonium carbamate from FTIR spectra. 

Lv et al. [14] irradiated ice mixtures including ammonia and carbon dioxide with 144 keV S(+9) cations. While simple warming seemed to produce ammonium carbamate and its dimer, irradiation produced N_2_O, OCN anion, and CO. Munoz-Caro and co-workers [15] directed 8.8 eV photons and also beams of 620 meV Zn(+26) and 19.6 meV Ne(+9) ions (emulating cosmic rays) towards methanol-ammonia ice. IR monitoring suggested production of carbonyl groups, perhaps from aldehydes, carboxylic acids, and esters.

Some experimental modeling investigations employ ices with three components. Vinogradoff et al. [16] employed an interstellar ice analog composed of water, CO_2_, ammonia, and formaldehyde. Bombardment with UV and ions produced a species identified as ammonium carbamate; carbamic acid was proposed as a catalyst. Noble and co-workers [17] studied several ices including HCN. Ammonium cyanide was identified as a predominant product. Esmaili and co-workers [18] irradiated carbon dioxide–methane–ammonia ices with electron beams with energy up to 70 eV, and attributed the production of glycine to low energy electrons

We note that all these studies rely on a few observed vibrational frequencies for the identification of specific molecules or at least functional groups, often in a condensed phase. A variety of computational models guided these assignments, but in no case is the reliability of the identification established.

## 2. Computational Modeling of Species Emerging from Water–Ammonia–Carbon Dioxide Ices

Modeling of species and processes occurring in ices presents considerable challenges, even beyond the study of orderly condensed phases. Woon [19] has discussed the special requirements of treating the chemistry and spectra of condensed phases. Since the experimental evidence summarized above for small molecules proposed to be formed in water–ammonia–carbon dioxide ices is derived from IR spectra of residues, computational modeling of those molecules without appreciation of the effects of the medium seems problematic. Modeling of carbamic acid and related species in their gas phase has therefore attracted considerable attention as a preliminary to more demanding studies of condensed phase mixtures. The complex of ammonia with carbon dioxide was first modeled by Amos et al. [20] by SCF methods, and later by Tsipis and Karipidis by DFT methods [21]. Remko and co-workers [22] have shown that carbamic acid is thermodynamically unstable relative to constituent NH_3_ and CO_2_ using SCF and MP2 theory as well as the CBS-QB3 thermochemical scheme. Ramachandran et al. [23] estimated a considerable (>50 kcal/mol) activation barrier to carbamic acid dissociation. Wen and Brooker [24] report SCF calculations on the zwitterionic form H_3_N(+)-CO_2_(−), finding it slightly less stable than the H_2_NCOOH form of carbamic acid in gas. The vibrational spectra for gas phase carbamic acid and the N,N–dimethyl variant have been evaluated in DFT models by Remko [25] and Jamróz and Dobrowski [26]. The 2:1 ammonia:carbon dioxide complex and the ammonia–carbamic acid complex have been similarly described [27]. Dell’Amico et al. [28] suggest that carbamic acid is implicated as a transient species accompanying the condensation of ammonia and carbon dioxide. Noble and co-workers [10] used B3LYP/6311G(d,p) to model the production of carbamate anion and carbamic acid in a cluster composed of a single CO_2_ and six ammonia molecules, concluding that those two products are comparably stable.

## 3. Plan of This Report

We first describe our methods for evaluation of structures and energies of candidate species in gas and condensed phases including ammonia, carbon dioxide, carbamic acid and its dimer, and 1:1 and 2:1 complexes of ammonia and carbon dioxide. Cations NH_4_^(+)^, the zwitterion of carbamic acid, and the salt ammonium carbamate are treated with special assumptions detailed below. All species reported so far as possibly present in CO_2_–NH_3_ ices are treated.

We establish a figure of merit that is intended to judge the quality of matching between observed and computed vibrational frequencies. A “match” is counted if an observed frequency falls within a frequency interval (FI) defined by the MP2 harmonic frequency and the frequency produced by anharmonic correction. We chose to define a FI for the following reason: harmonic frequencies computed by MP2 systematically over-estimate experimental values that include intrinsic anharmonicities. Anharmonic frequencies, however, typically underestimate experimental values. We suggest that the two computed frequencies establish a range (the FI) that should capture the physical system’s value. When an observed frequency falls within the FI, we award a point. If an observed value falls outside the computed FI by a few wave numbers, we award a half point. We use the figure of merit to judge reported frequencies from several experimental investigations.

## 4. Computational Methods for Structure and Energetics

For conventionally bound neutral species, we have chosen the thermochemical scheme W1BD [29] as implemented in the Gaussian 09 suite [30]. W1BD employs density functional models to obtain molecular geometry and vibrational frequencies and many-body corrections (Breuckner doubles) for correlation energy. The salt ammonium carbamate (NH_4_+)(H_2_NCOO-) and the zwitterionic form of carbamic acid H_3_N(+)COO(−) require special treatment. We first conducted W1BD calculations on separate ammonium cations and carbamate anions with no allowance for solvent, but of course medium effects are necessary for proper description of charged species in a condensed phase. For all systems, we estimated polarizable medium stabilization energies by ωB97XD/aug-cc-pVDZ calculations [31,32] with and without Tomasi’s model of a polarized continuum [33] with parameters for water using the Gaussian 09 suite. No specific interactions such as H–bonding were addressed. We estimated frequency shifts arising from the medium by CBSQB3 calculations using Gaussian 09 [30], again, with and without inclusion of the Tomasi.“water” medium.

## 5. Species Addressed

**Figure 1:**
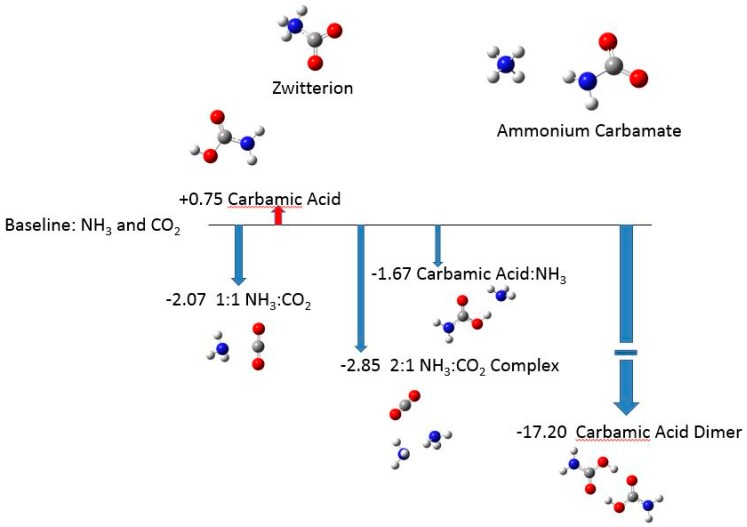
Species addressed in this report. Values are W1BD enthalpies in kcal/mol relative to equivalent numbers of fragments of NH_3_ and CO_2_.

Figure 1 shows the structures studied here. For later convenience, we introduce abbreviated names, including C for carbon dioxide and A for ammonia. Carbamic acid is called CA. Analogous abbreviated labels for other species are employed. The energy reference point is defined by the W1BD gas phase energy summed for sufficient carbon dioxide and ammonia molecules to account for all atoms in each structure. Thus, the reported energy is the gas phase binding energy of a species relative to those stable dissociation fragments. We see that conventionally represented carbamic acid (that is, not the zwitterion) in the gas phase is 0.75 kcal/mol less stable than CO_2_ + NH_3_. The zwitterionic form of carbamic acid (CA–Z) is considerably less stable without the stabilization of the condensed phase (ice), but the special assumptions made in the calculation prevent us from assigning a meaningful value for its relative energy. The same issue arises for the ammonium carbamate NH_4_+–H_2_NCO_2_− salt, called A(+):C(−). The W1BD energies put the unsolvated and separate ammonium and carbamate ions both at about 145 kcal/mol and the unsolvated zwitterion at about 36 kcal/mol above the unsolvated ammonia and carbon dioxide.

## 6. Results and Discussion

The reference fragments A and C can form a 1:1 A:C complex in vacuum, stabilized by 2.07 kcal/mol, or a 2:1 A:A:C complex stabilized by 2.85 kcal/mol. These structures feature a defining interaction between ammonia’s N and carbon dioxide’s C; the N…C interaction is comparable with a hydrogen bond in strength. Carbamic acid H_2_NCOOH is unstable by 0.75 kcal/mol. The complexation between carbamic acid CA and ammonia A forms CA:A. Forming the carbamic acid–ammonia species is stabilizing by 2.42 kcal/mol; the CA:A complex is 1.67 kcal/mol lower in energy than the stable fragments of carbon dioxide and two ammonias. The striking feature of our calculations is that the carbamic acid dimer (CA)_2_ is so strongly stabilized, lying 17.2 kcal/mol below the stable fragments of two carbon dioxide molecules and two ammonia molecules. These relative energies are displayed in Figure 1 (no medium correction).

## 7. Appreciation of Medium Effects: Stabilization Energies

Solvent corrections are not implemented in the W1BD suite, but we can get a first appreciation of such effects from simpler models. The medium stabilization energies shown in Table 1 are obtained from ωB97XD/aug-cc-pVDZ calculations with and without Tomasi’s model of a polarized continuum with parameters for water. Solvation has a large effect on charged species (the ammonium and carbamate ions) and highly dipolar species (the zwitterionic form of carbamic acid). However, at least in our approximate estimate, these species do not complete in stability with the dimer. Apart from the charged species, ammonium and carbamate and the zwitterionic form of carbamic acid, all effects are less than 10 kcal/mol. This, however, is sufficient to revise the profile of energies substantially.

**Figure 2:**
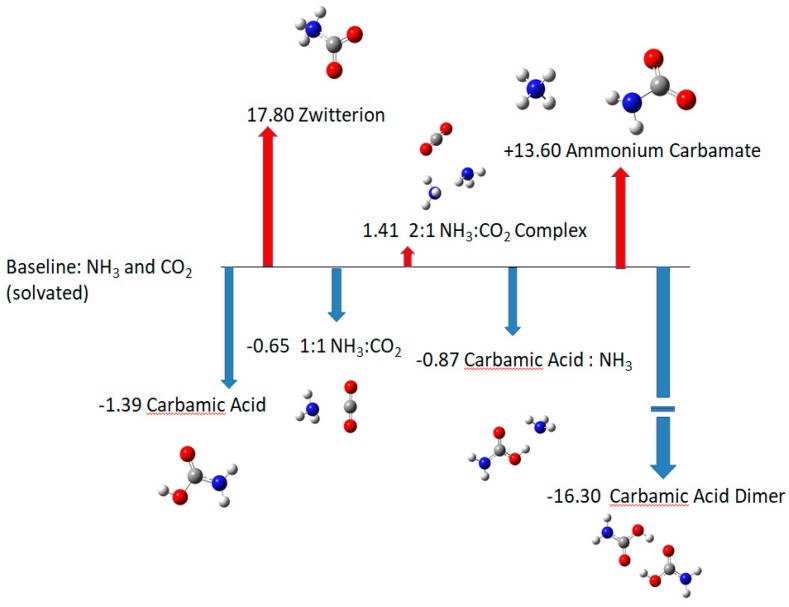
Relative enthalpies with estimated medium effect. Values in kcal/mol relative to equivalent numbers of ammonia and carbon dioxide molecules in medium.

Medium-adjusted enthalpies are shown in Figure 2. The carbamic acid monomer becomes more stable than fragments of ammonia and carbon dioxide. In contrast, the 2:1 ammonia:carbon dioxide complex is less stable than those dissociation products. The 1:1 ammonia:carbon dioxide complex is still stable, but the complexation of ammonia with carbamic acid is endoergic (requires energy input). The dimer is still much more stable than any alternative. Of course, formation of the dimer is disfavored by entropic factors if the abundance of carbamic acid is low.

## 8. Does the Medium Shift Frequencies Substantially?

We used the composite thermochemical scheme CBS-QB3 to judge the impact of the medium on computed harmonic frequencies. This method permits reoptimization of each structure in response to the medium, and estimates frequency shifts arising from both structure changes and the effective potential as corrected for the presence of the polarizable medium. These calculations show that, most often, frequency shifts are minor (less than 20 cm^−1^). Some corrections are more serious: the leading example is the carbamic acid–ammonia complex, for which the H_3_N…H–O hydrogen bond is strengthened and shortened (and the OH stretch is red shifted) and the H_2_NH…O= bond is weakened. The vibrational frequencies of the zwitterionic form of carbamic acid are systematically red-shifted, by about 3–5%. The carbamic acid dimer shows medium-induced red shifting of about 50 cm^−1^ for the out-of-plane motion of OH bonds near 950 cm^−1^ and also for the composite motion combining the OCO asymmetric stretches and the COH bends near 1750 cm^−1^. More typically, the response to the medium is minor for frequencies above 500 cm^−1^; this is the case for even the weakly bound 1:1 A:C and 2:1 A:A:C_2_ complexes, for which shifts are no more than 20 cm^−1^. Details are compiled in the supplementary information.

## 9. Are Carbamic Species Distinguishable by Computed Vibrational Spectra?

We use the independently calculated MP2/aug-cc-pVDZ//W1BD (no medium correction) harmonic frequencies with anharmonic corrections obtained by the Gaussian 09 suite to judge the assignments to structures in experimental reports. Our comparison is between “frequency intervals” defined as the range between harmonic and anharmonic computed frequencies. Specifically, as shown in Table 2, the highest frequency FI is 3563 to 3721 cm^−1^ for the monomer, while the highest frequency FI is 3474 to 3631 cm^−1^ for the 1:1 complex A:C or H_3_N:CO_2_. The two FIs overlap, so we count a match (shown as “YES” in Table 2.) We define a figure of merit, or matching fraction, as the ratio of experimental frequencies matched in the range MP2 (blue extreme) to MP2 + anharmonic corrections (red extreme). Our first exercise is to see how similar the MP2 computed frequency ranges are among the several species. Seemingly, structurally distinct molecules with high similarity would be hard to distinguish by their vibrational spectra. Entries are percentages of FIs common to two molecules. Of course, the diagonal values in this table must be 100%, but totally disjointed sets of FIs could produce a score of zero.

Here is how we arrive at a value for a similarity index. Consider the computed MP2 frequencies for the carbamic acid monomer and for the 1:1 ammonia–carbon dioxide complex. Computed MP2 frequencies in harmonic approximation and anharmonic corrections are reported in Table 2. Consider the Monomer’s first frequency interval (FI), from 3721 to 3563 cm^−1^. This FI overlaps with the first FI for the 1:1 complex, so we could recognize one point of agreement. The second FI for the monomer (3823 to 3648 cm^−1^) lies slightly outside any FI for the 1:1 complex. We could perhaps award half a point for the near miss. The FI 3599 to 3456 cm^−1^ finds a match in the 1:1 complex set, so it is given one full point. Continuing to the FI of the monomer 1854 to 1814 cm^−1^, we find no counterpart in the 1:1 complex set of FIs. The FI 1647 to 1602 cm^−1^ does overlap with the FIs of both complexes 1648 to 1602 cm^−1^ and 1648 to 1589 cm^−1^.

Continuing in this fashion, we find 6.0 points of agreement in the 15 FIs for the carbamic acid monomer, so the similarity index for the 1:1 complex to the monomer is 6.0/15 or 40%. If we set aside the near misses, we find 4/15 points of agreement (27%).

Comparisons among computed FIs are collected in Table 3. A sample entry can be read as the percent of all FIs computed for [row label] found in the set computed for [column label]. More specifically, of all 28 FIs computed for (CA)_2_, 60% are found in the FIs computed for the complex CA:A. In contrast, of the FIs computed for (CA)_2_, only 27% are to be found in the computed FIs for the 1:1 complex A:CO_2_, while 88% are to be found in the 1:1 carbamic acid–ammonia complex CA:A. (These entries are bold in Table 3.) Unsurprisingly, the greatest degree of similarity is between the 1:1 complex of ammonia with carbon dioxide and the 2:1 complex of ammonia with carbon dioxide. These results illustrate a high degree of similarity among all proposed species, and serve as a precaution against overconfident attribution of observed frequencies to particular species.

Intensities and response of frequencies to isotopic substitution could enhance discrimination. Experimental traces allow at least semiquantitative evaluation of intensities. Inferences would be complicated by the fact that observed intensities are affected by both the intrinsic properties of the mode and the abundance of the species in question.

While to our knowledge no data on isotope effects have been included in reports of experimental studies of synthetic ices, nothing in principle prevents such refinements. To provide a first impression of possible results, we report computed harmonic frequencies for the most intense vibrational transitions for the set of candidates (supplementary information). Those modes likely to show substantial shifts upon perdeuteration are identified. For species incorporating the carboxyl group and a coordinated NH_3_, we expect major effects on the OH and NH stretching regions, with shifts approaching the limit of 1/√(2). Lesser effects are to be observed for bends involving H(D) atoms. Perdeuteration may allow discrimination between closely related species, notably the complexes of carbon dioxide with one and two ammonia molecules. Perdeuteration seems to have a notable impact only on the 1100 cm^−1^ NH_3_ pyramidalization band of the carbon dioxide–ammonia complex, while many more of the modes of the CO_2_(NH_3_)_2_ complex are affected.

## 10. Fingerprint Regions?

Of course our method of frequency interval matching is not how IR spectra are ordinarily used to discriminate between or among species in a mixture. One looks for distinct absorption frequencies by which specific structures can be recognized. Ideally. such descriptive features should be intense and easily detected. Table 4 shows computed FIs for a number of strong and moderately intense IR features. For example, among the computed FIs for the monomer, we find one FI (1854 to 1814 cm^−1^) intensely absorbing and unique to the monomer. Another FI of the monomer (1239 to 1210 cm^−1^) is close to FIs for the ammonium carbamate salt and the A:CO_2_ 1:1 complex. As the detailed tables in the supplementary information show, it is often the case that the most distinctive FI for a particular structure is matched by at least one FI for another species in the set. For example, there are no distinctive FIs uniquely present for the 1:1 complex. The FI 2380 to 2341 cm^−1^ for the 1:1 complex is the most characteristic, held in common only by the 2:1 A:A:CO_2_ complex. This is to be expected from the very similar structure of the 1:1 and 2:1 complexes. In this case, we offer no prospect of distinguishing the 1:1 from the 2:1 complex by their IR spectra.

Still, the information contained in the computed spectra may be sufficient to exclude certain structures from the list, and perhaps assign probabilities to the presence of specific forms. Consider the CA dimer: its computed FI 1498 to 1456 finds a counterpart in the FIs for the carbamic acid complex with ammonia, 1495 to 1432. Two frequencies of the dimer ca. 1380–1350 cm^−1^ are distinguishable from any FI in the complex, as is another FI from 550–564 cm^−1^. These FIs can serve as fingerprint regions of the spectra.

## 11. Is It Feasible to Attribute Observed Frequencies to Specific Species?

It is certainly tempting to associate observed frequencies with plausible structures, and investigators have not resisted the temptation. Here, we review several attributions and establish similarity scores for those proposals. Full details and instructions for scoring are in the supplementary information.

Khanna and Moore [12] report IR frequencies for species appearing in the condensed residue after irradiation. They assign a number of IR bands to ammonium carbamate and others to the zwitterionic form of carbamic acid. Detailed compilations of the frequencies observed by Khanna and Moore [12] and the frequency intervals (FIs) computed for each of our seven species are provided in the supplementary data. We consider all 31 frequencies observed and reported by Khanna and Moore [12]. Scoring in the same way as we evaluated matches among computed spectra, we find that the computed fundamental frequencies score 14.5 for the carbamic acid–ammonia complex; 11.5 for carbamic acid dimer, 11.0 for ammonium carbamate, and 9.5 for each of the three other structures, i.e., the zwitterionic carbamic acid and the 1:1 and 2:1 ammonia–carbon dioxide complexes. No single species accounts for as much as half of the observed frequencies. If we include the leading candidate and add the carbamic acid–ammonia complex, and also ammonium carbamate, the score rises to 19, or 61% of the reported observed frequencies. None of the remaining species add so substantially to the match score. For example, the score for the carbamic acid–ammonia complex plus the carbamic acid dimer rises only to 17 (55%). The implication is that one should include the carbamic acid–ammonia complex in the discussion of ammonia–carbon dioxide ices.

Why does it seem impossible to match all reported vibrational frequencies with computed FIs? We compute only fundamental frequencies, while experimental reports may include overtones and combination bands in the set of observed frequencies. There are many low-frequency modes in these species, and indeed our calculations of anharmonicity predict overtones and combinations, some of which have reasonably large intensity. We have not included these frequencies in our match scores, thinking that the general outcome would be indicated in the first stage of the analysis.

The 15 frequencies reported by Hisatsune [5] also suggest that the carbamic acid–ammonia complex is significant. It receives the highest score (7.5), while the ammonium carbamate salt earns 7.0; other structures lag. The combination of the complex and the salt earns a 10.0 score.

Bossa, et al. [7,8] observe 15 frequencies, which they assign to ammonium carbamate and the dimer of carbamic acid. The leading score of 10.5 is earned by the carbamic acid–ammonia complex, while ammonium carbamate is second with 7.5 points. Considering both the complex and the salt increases the total score to 12.0 points. The dimer by itself scores 6.5 points while the combination of dimer and salt totals 12.0 points. The set of three species complex, salt, and dimer does not increase the score further. Deciding between the presence of CA:A complex and (CA)_2_ dimer requires closer attention.

Chen et al. [6] report five frequencies, and conclude that the carbamic acid monomer is present owing to the observed absorption at 1720 cm^−1^, assigned to COO asymmetric stretching. Calculations conflict with this assignment, however. DFT calculations (scaled by a factor of 0.98) and MP2 calculations with anharmonicity corrections put this motion at about 1800 cm^−1^. Lower frequencies near 1720 cm^−1^ are calculated for ammonium carbamate, the dimer, and the complex of carbamic acid with ammonia. The best match (score 4.0) with the five frequencies reported by Chen et al. [6] is found for the carbamic acid:ammonia complex.

Rodriguez-Lazcano et al. [9] report 17 frequencies at 140 K and 20 frequencies at 240 K from their vapor deposition studies (we do not include the “shoulders”). They infer the presence of ammonium carbamate and offer evidence for a carbamic acid species. They suggest that the species could be isolated carbamate anion, without ruling out the possibility of any or all of neutral, zwitterionic, and dimeric carbamic acid species. Our matching exercise for the high temperature data shows the carbamic acid–ammonia complex to be the leading single candidate at high temperature with a score of 12, followed by the dimer (10) and ammonium carbamate (9.5). The best score for two species is 16.5 for the dimer and ammonium carbamate, followed by 15.5 for ammonium carbamate and the carbamic acid–ammonia complex. The low-temperature data also suggest the presence of ammonium carbamate and allow the possibility of either or both the dimer and the ammonia–carbamic acid complex.

Noble et al. [10] report 14 frequencies in an ammonia–carbon dioxide ice between 70 and 90 K. They suggest that ammonium carbamate is a major product of the warming, and assign some frequencies to carbamic acid and to its dimer. In our scoring the carbamic acid–ammonia complex achieves a score of 10, followed by ammonium carbamate (8.5) and then the carbamic acid dimer and the 2:1 ammonia–carbon dioxide complex. The best score for a two-component system includes the carbamic acid–ammonia complex and ammonium carbamate, totaling 12.

## 12. Conclusions

Ammonium carbamate and the carbamic acid complex with ammonia seem to be the most likely products of irradiation of ammonia–carbon dioxide ices. The carbamic acid dimer is also a candidate, but the abundance of ammonia in most of the experimentally studied solid environments also points to production of the ammonia complex. The pathway for conversion of ammonium carbamate to the ammonia–carbamic acid complex in the solid state is worth exploring. We recommend that investigations of models of prebiotic synthesis of small molecules and astronomical data include consideration of the possibility of the ammonia–carbamic acid complex.

## Figures and Tables

**Table 1:** Solvation effects on relative energies of carbamate species.

Species	CA	(CA)_2_	Z	NH_4_(+) and Carbamate(-)	NH_3_:CO_2_	2NH_3_:CO_2_	Carbamic Acid:NH_3_	NH_3_	CO_2_
Medium E (kcal/mol)	−6.68	−8.16	−23.01	−139.03	−3.11	−3.58	−6.77	−3.04	−1.50
Relative to solvated NH_3_ and CO_2_	−1.39	−16.30	17.80	13.24	−0.65	+1.14	−0.87	R-0-	-0-

**Table 2:** Computed frequency intervals (frequencies in wave numbers) for two carbamic system species and the match index indicating that of the 15 frequency intervals (FIs) calculated for the carbamic acid monomer; counterparts for six observed frequencies can be found in the set of FIs calculated for the 1:1 ammonia:carbon dioxide complex.

Monomer M:H_2_N–COOH		Match to M Found in 1:1 Spectrum?	1:1 Complex H_3_N:CO_2_	
Harmonic	Anharmonic		Harmonic	Anharmonic
3721	3563	Yes (FIs overlap)	3631	3474
3823	3648	Maybe (FIs almost touch)	3629	3464
3599	3456	Yes (FIs overlap)	3477	3328
1854	1814	No		
1647	1602	Yes (FIs almost coincide)	1648	1602
1373	1322	Maybe (FIs almost touch)	1308	1248
1239	1210	Maybe (FIs almost touch)		
1094	1066	Yes (FIs overlap)	1076	1005
945	921	No		
776	745	No		
609	615	Maybe (FIs almost touch)	634	652
566	429	No		
521	513	No		
446	401	No		
341	260	No		
Total FI	15	6 (40%) accounted		

**Table 3:** Similarity indices for computed vibrational frequencies; maximum value is 100. These values include half point values for near-misses. Entries in bold face are discussed in the text.

Similarity	CA	CA–Z	A:CO_2_	A:A:CO_2_	A+:C−	CA:A	(CA)2
CA (15)	-	43	40	43	63	60	57
CA–Z (12)	42	-	33	33	50	71	58
A:CO_2_ (10)	60	50	-	95	80	70	60
A:A:CO_2_	79	38	94	-	59	76	62
A+:C− (15)	63	40	43	70	-	77	43
CA:A	64	59	52	67	59	-	88
(CA)_2_ (28)	**57**	52	**27**	**27**	45	**88**	-

**Table 4:** Most prominent features of the computed vibrational spectra (frequencies in wave numbers; frequency intervals computed by MP2/aug-cc-pVTZ).

Species	Strong Transitions (FI)	Medium-Strength Transitions (FI)
NH_3_ CO_2_ complex	None	2380−2341
(NH_3_)_2_ CO_2_ complex	None	2381−2343
Monomer	1854–1814	1647–1602; 1373−1322; 1239−1210
Dimer	3163–2783; 1763–1714; 1626–1584; 1385–1355	3786–3514; 976–951
Zwitterion	1807–1788; 1443−1389	
Ammonium carbamate	1670–1639	3646–3346; 1308–1268; 827–797; 665–630
Carbamic acid–NH_3_ complex	3108–2757; 1779–1738; 1361–1317	1612–1557; 1115–1059

## Supplementary Materials

The following are available online at https://www.mdpi.com/2075-1729/9/2/34/s1, Coordinates of all species, detailed description of evaluation of figure of merit MATCH for all comparisons.
